# Real-World Outcomes of Pralsetinib in RET Fusion-Positive NSCLC

**DOI:** 10.1016/j.jtocrr.2024.100743

**Published:** 2024-10-17

**Authors:** Francesca Lucibello, Valérie Gounant, Mihaela Aldea, Michaël Duruisseaux, Maurice Perol, Christos Chouaid, Jaafar Bennouna, Vincent Fallet, Aldo Renault, Florian Guisier, Etienne Giroux-Leprieur, Marie Wislez, Anne-Claire Toffart, Julien Mazieres, Clémence Basse, Nadia Hegarat, Matthieu Carton, Nicolas Girard

**Affiliations:** a Institut Curie, Institut du Thorax, Paris, France; b Université Paris Cité, Service d’Oncologie thoracique & CIC1425 INSERM, Hôpital Bichat Claude Bernard, AP-HP. Nord, Paris, France; c Gustave Roussy, Oncologie médicale, Villejuif, France; d Hospices Civils de Lyon, Hôpital Louis Pradel, Bron, France; e Centre Léon Bérard, Lyon, France; f CHIC, Service de Pneumologie, Créteil, France; g Hôpital Foch, Oncologie Médicale, Suresnes, France; h APHP, Service de Pneumologie, Hôpital Tenon, Paris, France; i CH, Service de Pneumologie, Pau, France; j Normandie Univ, UNIROUEN, LITIS Lab QuantIF team EA4108, CHU Rouen, and Inserm CIC-CRB 1404, Rouen, France; k APHP, Service de Pneumologie, Hôpital Ambroise Paré, Boulogne, France; l APHP, Service de Pneumologie, Hôpital Cochin, Paris, France; m CHUGA, Service de Pneumologie, Grenoble, France; n CHU Toulouse, Service de Pneumologie, Toulouse, France; o Paris Saclay, UVSQ, UFR Simone Veil, Versailles, France

**Keywords:** Pralsetinib, Lung Cancer, RET, Second-Line

## Abstract

**Introduction:**

Pralsetinib is a RET inhibitor found to have antitumor activity in advanced, metastatic, *RET* fusion-positive NSCLC.

**Objective:**

To assess real-world efficacy of pralsetinib and treatment sequences in patients with RET fusion-positive NSCLC.

**Design:**

Retrospective study of consecutive patients enrolled in the French expanded-access program for pralsetinib from December 1, 2019, to December 31, 2021.

**Participants:**

A total of 41 patients with advanced, refractory, *RET* fusion-positive NSCLC were included. Pralsetinib was administered at a daily dose of 400 mg based on safety and pharmacokinetic outcomes from previous phase 1/2 study.

**Results:**

Pralsetinib was administered as second line in 23 patients (56%) and as third line and beyond in 15 patients (37%). After a median follow-up of 26.3 months, pralsetinib was ongoing in 13 patients. Median real-world progression-free survival was 11.8 (95% confidence interval [CI]: 9.3–15.5) months. Objective response rate was 68% (95% CI: 50%–82%), and disease control rate was 89% (95% CI: 75%–97%). Subsequent line of systemic therapy was initiated in 11 patients. Median overall survival from pralsetinib initiation was 23.8 (95% CI: 16.5–not reached) months.

**Conclusion:**

In this extensive real-world cohort of patients with advanced or metastatic NSCLC harboring RET fusions, we highlight the antitumor efficacy of pralsetinib, particularly when administered in later treatment lines. We also observe the aggressive nature of disease progression, frequent utilization of chemotherapy and antiangiogenic agents as initial subsequent therapies, and limited insight into resistance mechanisms due to infrequent rebiopsy and genomic profiling at progression.

## Introduction

*RET*, gene fusions are observed in 1% to 2% of NSCLC and represent a target for precision medicine strategies using RET inhibitors.^1^^,^^2^ Testing for *RET* fusions, to give treatment with available highly selective inhibitors which include selpercatinib and pralsetinib, is standard of care in the most recently published clinical practice guidelines.^3^^,^^4^ From the ARROW phase 1/2 trial in 281 patients with *RET* fusion-positive NSCLC, pralsetinib was found to have an objective response rate (ORR) of 59% (95% confidence interval [CI]: 50%–67%) and a median progression-free survival (PFS) of 16.5 months (95% CI: 10.5–24.1) in pretreated patients.^5^ Although these agents are being integrated in the first-line setting,^6^ there is a need to better understand not only their real-world efficacy but also, more importantly, which treatment sequences are administered in *RET* fusion-positive NSCLC.

## Methods

### Study Design and Participants

This is a retrospective study of all consecutive patients enrolled in the French expanded-access program for pralsetinib from December 1, 2019, to December 31, 2021. Eligible patients were aged 18 years or older who had unresectable, locally advanced, or metastatic NSCLC with a documented *RET* fusion. Pralsetinib was administered daily at a dose of 400 mg once a day.^5^ The study was conducted in accordance with MR-004 regulation of the French National Agency regulating Data Protection.

### Outcomes

The primary end point was real-world PFS and secondary end points were investigator-assessed ORR per Response Evaluation Criteria in Solid Tumors criteria considering confirmed responses, disease control rate, intracranial PFS, and overall survival (OS) from pralsetinib initiation.

## Results

### Patient Population

A total of 41 patients were included at 25 participating centers. There were 16 men (39%), 20 never-smokers (49%), and all had histologically confirmed, advanced or metastatic NSCLC (Table 1). Most had *KIF5-RET* (18 cases, 44%) or *CCDC6-RET* (six cases, 15%) fusion; genomic profiling at time of diagnosis led to the simultaneous identification of the following co-alterations in three patients (9%): *EGFR* mutation in one patient, *KRAS* mutation in one patient, and *ALK* rearrangement in one patient.

**Table 1:** Baseline Clinical Characteristics of Patients

Characteristics	N = 41, n (%)
Age, y	
<60	22 (54)
>60	19 (46)
Sex	
Male	16 (39)
Female	25 (61)
Smoking status	
Never smoked	20 (49)
Former smoker	18 (44)
Current smoker	3 (7)
Histology	
Adenocarcinoma	37 (93)
Others	4 (7)
Stage	
IIIb	4 (10)
IV	35 (85)
Missing data	2 (5)
ECOG performance status^a^	
0	6 (16)
1	24 (63)
2	7 (18)
Missing data	4 (3)
Metastasis at diagnosis	
Lung	13 (32)
Liver	7 (17)
Bone	18 (44)
Brain	4 (10)
RET-fusion results^b^	
*KIF5-RET*	18 (44)
*CCDC6-RET*	6 (15)
Others^c^	3 (7)
Unknown	5 (12)
Missing data	9 (22)
PD-L1, %	
<1%	2 (5)
1%–49%	13 (32)
>50%	10 (24)
Missing data	16 (39)
Line of treatment	
1	3 (7)
2	23 (56)
3	6 (15)
>4	9 (22)

ECOG, Eastern Cooperative Oncology Group; PD-L1, programmed death-ligand 1.

a ECOG performance status scores range from 0 to 5, with higher numbers reflecting greater disability.

b Technique: Fusion status was assessed by multiple techniques (ARN, and, and FISH).

c In this category, *RET* fusion was founded by molecular analysis but the *RET* fusion partner was not identified.

**Figure 1:**
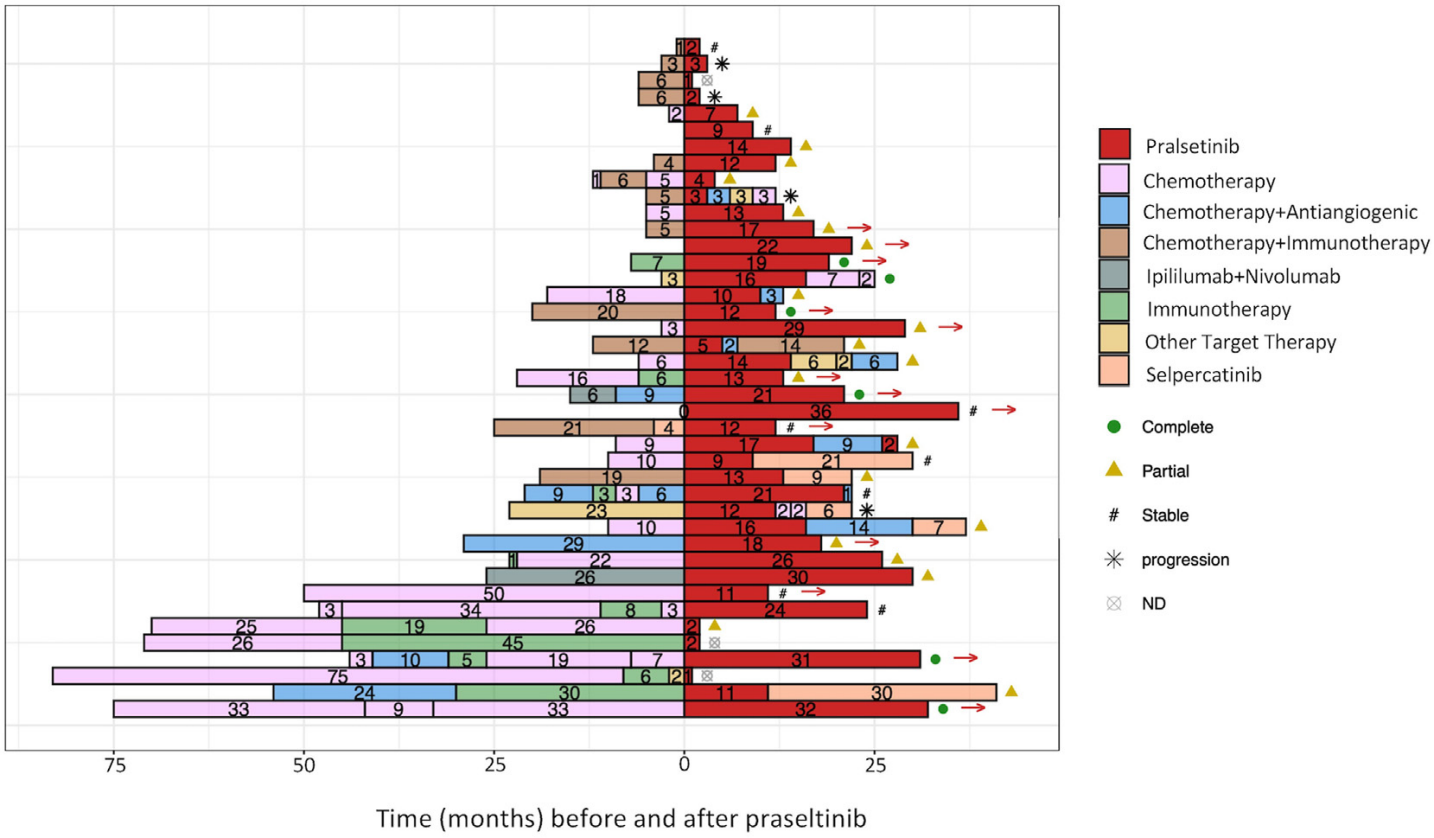
Treatment sequences before and after pralsetinib.

Pralsetinib was administered as second line in 23 patients (56%) and as third line and beyond in 15 patients (37%) (Fig. 1); 33 (88%) had previously received platinum-based chemotherapy, associated with immune checkpoint inhibitors in 11 cases and with bevacizumab in three cases. Median duration of previous lines was 9 months. Two patients with *EGFR*-mutated and *ALK*-rearranged tumor had received tyrosine kinase inhibitors previously.

### Efficacy of Pralsetinib

**Figure 2:**
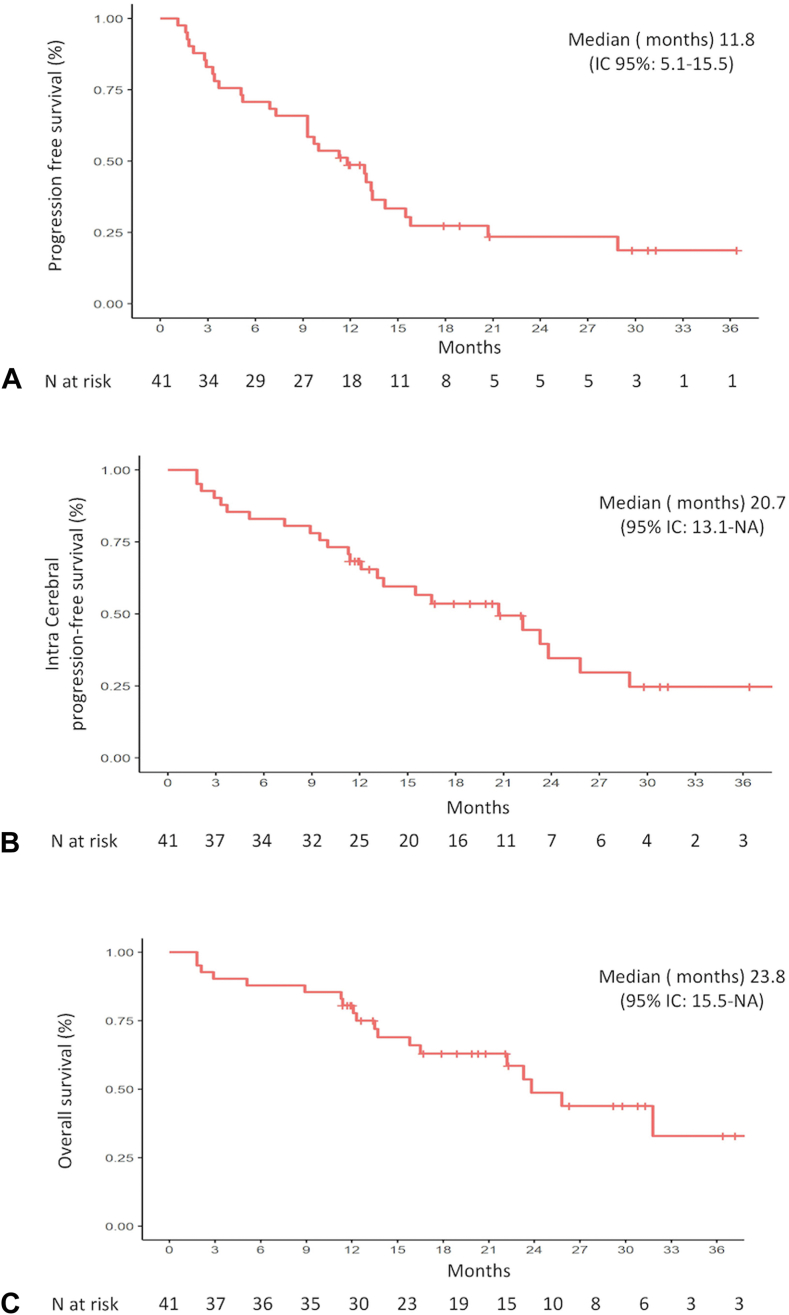
Kaplan-Meier analyses of (*A*) progression-free survival, (*B*) intracranial progression-free survival, and (*C*) overall survival.

ORR was 68% (95% CI: 50%–82%) and disease control rate was 89% (95% CI: 75%–97%). Intracranial response was observed in four patients with active brain metastases at baseline; of note, none of these had received concurrent brain radiotherapy. After a median follow-up of 26.3 (95% CI: 20.3–36.4) months, 30 patients experienced disease progression, five of whom died; pralsetinib was still ongoing in 13 patients, including two patients treated beyond progression. Median PFS was 11.8 (95% CI: 9.3–15.5) months (Fig. 2*A*). PFS did not significantly differ depending on Eastern Cooperative Oncology Group performance status, line of treatment, type of previous therapies, or metastatic sites. PFS was 12, 16, and 36 months in patients with *EGFR*, *ALK*, and *KRAS* co-alterations, respectively.

Most frequent sites of disease progression included the lung (40% of patients), brain (38% of patients), bones (21% of patients), and pleura (16% of patients); only two patients had oligoprogression. Median intracranial PFS was 21 (95% CI: 13.1–not reached) months (Fig. 2*B*).

### Safety of Pralsetinib

Overall, 22 patients (54%) experienced at least one grade greater than or equal to 3 treatment-related adverse event. Grade 3 toxicity was observed in 16 patients (41%), consisting of increased creatine phosphokinase levels, hematological events (neutropenia or anemia), hypertension, and interstitial pneumonitis in two patients—with an immediate previous exposure to immune checkpoint inhibitors. Grade 4 adverse events were interstitial pneumonitis in one patient, again with immediate previous exposure to immunotherapy, and neutropenia in one patient. For pneumonitis, the actual relation with pralsetinib may be questioned, as these events may have been driven by immunotherapy. These adverse events led to pralsetinib interruption in 16 patients (39%) and dose reduction in 10 patients (24%).

### Treatment Sequences and OS

Among the 23 patients who experienced disease progression and discontinued pralsetinib, subsequent therapy was delivered in 11 patients; no patient had comprehensive genomic profiling performed. Subsequent therapies included paclitaxel-bevacizumab with or without carboplatin regimen in five patients, carboplatin-pemetrexed or gemcitabine in one patient each, selpercatinib in three patients, and crizotinib in one patient. Median duration of first subsequent line of systemic therapy was 8 months for chemotherapy and 8.5 months for selpercatinib.

Median OS from pralsetinib initiation was 23.8 (95% CI: 16.5–not reached) months (Fig. 2*C*). OS did not significantly differ with respect to performance status, line of treatment, or metastatic sites.

## Discussion

From this large real-world cohort of patients with *RET* fusion-positive, advanced or metastatic NSCLC, we report the following: (1) high antitumor activity of pralsetinib delivered in the late line setting; (2) the aggressiveness of disease progression on pralsetinib; and (3) the poor understanding of resistance mechanisms given infrequent rebiopsy and genomic profiling at progression.

In our cohort, pralsetinib was administered as second line in 23 patients (56%) and as third line and beyond in 15 patients (37%), and we report an ORR of 68% and disease control rate of 89%, with PFS rates at 6 and 12 months of 71% and 49%, respectively. These results are in line with those reported in the ARROW phase 1/2 trial,^5^ although median PFS in our cohort—11.8 months—was lower.^5^ Median intracranial PFS was 21 months in our cohort, highlighting the major weight of central nervous system disease control in the overall PFS. Noticeably, most patients had previously received platinum-based chemotherapy possibly combined with immunotherapy, which is in line with the control arm of the first-line LIBRETTO-431 trial.^6^ Ultimately, efficacy in our cohort was also in line with that reported from the Korean expanded-access program cohort—response rate of 57% and median PFS of 12.1 months.^7^

In our study, most patients presented with multisite disease progression after pralsetinib. This is also highlighted in recent cohorts.^8^ A better understanding of the molecular mechanisms underlying sensitivity and resistance to RET inhibitors, is clearly warranted.^9^ Another finding in our cohort is the high frequency of co-alterations in *RET* fusion-positive NSCLC, with 9% of cases diagnosed concurrently with *EGFR*, *KRAS*, and *ALK* alterations, which highlights the need for comprehensive genomic profiling even if such a more frequent alteration is identified, not to miss a *RET* fusion leading to potential eligibility to RET inhibitors; in our cohort, these patients had sustained response to pralsetinib. Such co-mutations have not been described so far in the literature, excluding cases of emergent *RET* fusions as an acquired resistance mechanism to tyrosine kinase inhibitors.^10^

In our cohort, no patients underwent rebiopsy at the time of disease progression after treatment with pralsetinib. This may be due to the high tumor burden of progressing sites, which were multiple and potentially life-threatening. Liquid biopsy could be a useful tool in this context; however, recent studies suggest that tissue biopsy is necessary due to potential histologic transformation.^11^ A multi-institutional analysis of repeat tumor or plasma biopsies from patients with *RET* fusion-positive NSCLC treated with selpercatinib and pralsetinib identified the emergence of *MET* amplification (15%) and *KRAS* amplification in one case each.^12^ Continuous assessment of resistance mechanisms in larger cohorts of *RET*-altered tumors is essential to develop next-generation RET inhibitors and combination therapies to overcome resistance.

Ultimately, RET inhibitors are currently being positioned as first-line therapy for *RET* fusion-positive NSCLC. Although the AcceleRET trial with pralsetinib is still ongoing (NCT04222972), the LIBRETTO-431 randomized, phase 3 trial recently revealed a significant PFS benefit with selpercatinib when compared with platinum-based chemotherapy with or without pembrolizumab, as first-line therapy in 212 patients^6^; median PFS was 24.8 months versus 11.2 months (hazard ratio = 0.46, 95% CI: 0.31–0.70, *p* < 0.001), respectively. At interim analysis, no data were released regarding subsequent therapies delivered after selpercatinib. Our study provides evidence of the feasibility and potential efficacy of platinum-based chemotherapy associated with bevacizumab as first subsequent therapy after RET inhibitor treatment.

To conclude, our data highlight the efficacy of pralsetinib in a real-word setting and the potential treatment sequences in patients with RET fusion-positive NSCLC.

## CRediT Authorship Contribution Statement

**Francesca Lucibello:** Conceptualization and writing, Final approval of the manuscript.

**Valérie Gounant:** Resources, Final approval of the manuscript.

**Mihaela Aldea:** Resources, Final approval of the manuscript.

**Michaël Duruisseaux:** Resources, Final approval of the manuscript.

**Maurice Perol:** Resources, Final approval of the manuscript.

**Christos Chouaid:** Resources, Final approval of the manuscript.

**Jaafar Bennouna:** Resources, Final approval of the manuscript.

**Vincent Fallet:** Resources, Final approval of the manuscript.

**Aldo Renault:** Resources, Final approval of the manuscript.

**Florian Guisier:** Resources, Final approval of the manuscript.

**Etienne Giroux-Leprieur:** Resources, Final approval of the manuscript.

**Marie Wislez:** Resources, Final approval of the manuscript.

**Anne-Claire Toffart:** Resources, Final approval of the manuscript.

**Julien Mazieres:** Resources, Final approval of the manuscript.

**Clémence Basse:** Resources, Final approval of the manuscript.

**Nadia Hegarat:** Project administration, Final approval of the manuscript.

**Matthieu Carton:** Methodology and formal analysis, Final approval of the manuscript.

**Nicolas Girard:** Conceptualization, Resources, Writing, Visualization, Funding acquisition, Final approval of the manuscript.

## Disclosure

Dr Gounant reports consultancy for AstraZeneca, Bristol Myers Squibb, Janssen, Pfizer, Sanofi, Takeda, Roche. Hospitality from Jansse, Pfizer, Sandi, Takeda, and Roche. Dr Aldea reports research funding from Sandoz, AstraZeneca, and Amgen; advisory board for Viatris. Dr Perol reports consultancy for BMS, MSD, Astra Zeneca, Roche, Daiichi Sankyo, Janssen, Ipsen, Esai, GSK, Eli Lilly, Pfizer, Takeda, and Novocure; lectures for BMS, MSD, Astra Zeneca, AnHeart, Sanofi, Pfizer, Takeda, and Janssen; hospitality from BMS, MSD, Astra Zeneca, Roche, Pfizer, Takeda; DSMB for Roche, and Pharmamar. Dr Guisier reports consulting or lectures from Amgen, Astra Zeneca, BMS, Sanofi, Viatris, Takeeda, Roche, MSD, Pfizer, and Janssen; reports research grants to institution from Takeda, Roche, and Pfizer, outside of the submitted work. Dr Mazieres reports consultancy for Merck, Astra Zeneca, BMS, MSD, Roche, Novartis, Daiichi Sankyo, and Pfizer. Dr Girard reports research grants/support from Abbvie, Amgen, AstraZeneca, Beigene, Boehringer Ingelheim, Bristol Myers Squibb, Daiichi-Sankyo, Gilead, Hoffmann-La Roche, Janssen, LeoPharma, Lilly, Merk Serono, Merck Sharp & Dohme, Novartis, Sanofi, and Sivan; serving on the consultative services for Abbvie, Amgen, AstraZeneca, Beigene, Bristol Myers Squibb, Daiichi-Sankyo, Gilead, Ipsen Hoffmann-La Roche, Janssen, LeoPharma, Lilly, Merck Sharp & Dohme, Mirati, Novartis, Pfizer, Pierre Fabre, Sanofi, and Takeda; participation on a data safety monitoring board for Hoffmann-La Roche; employment of a family member with AstraZeneca.
